# Emerging Topics in Metal Complexes: Pharmacological Activity

**DOI:** 10.3390/ijms25094982

**Published:** 2024-05-03

**Authors:** Agnieszka Ścibior, Manuel Aureliano, Juan Llopis

**Affiliations:** 1 Laboratory of Oxidative Stress, Department of Biomedicine and Environmental Research, The John Paul II Catholic University of Lublin, 20-708 Lublin, Poland; 2 Faculdade de Ciências e Tecnologia (FCT), Campus de Gambelas, Universidade do Algarve, 8005-139 Faro, Portugal; 3 Centro de Ciências do Mar (CCMar), Campus de Gambelas, 8005-139 Faro, Portugal; 4 Institute of Nutrition and Food Technology “José Mataix Verdú”, Department of Physiology, Biomedical Research Center, University of Granada, Avda del Conocimiento s/n., 18100 Armilla, Granada, Spain; jllopis@ugr.es

## 1. Introduction and Scope

**Figure 1:**
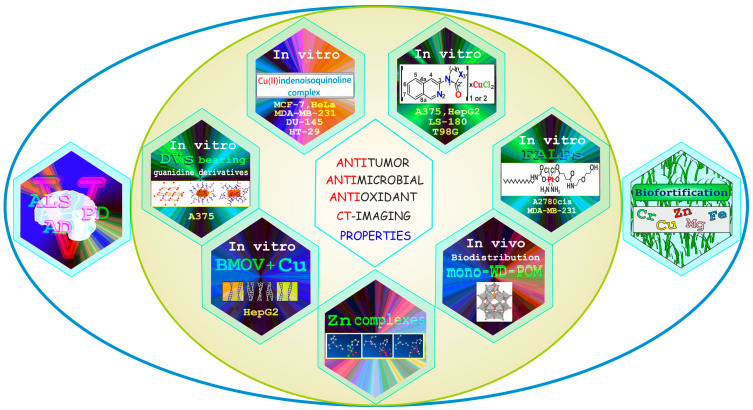
Graphical summary of metals and metal compounds/complexes overviewed in this Special Issue: neurodegenerative disorders, in vitro and in vivo studies, anticancer and antimicrobial activity, antioxidant and CT-imaging properties, biodistribution, and biofortification.

This Special Issue (SI), ”Emerging Topics in Metal Complexes: Pharmacological Activity”, includes reports updating our knowledge on metals with multidirectional biological properties and metal-containing compounds/complexes for their potential therapeutic applications, with a focus on strategies improving their pharmacological features. The current SI also refers to the biofortification process, which, in the near future, may become a promising source of dietary enrichment with elements important for human health. The scientific articles making up the current SI, i.e., one review and eight original papers (nine in total), garnered a total of six citations and over 8600 visualizations (April 2024). A graphical summary of the content included in this Special Issue is presented in Figure 1, showing a central image with seven hexagons reflecting the seven papers on antitumor, antimicrobial, antioxidant, and CT-imaging properties, flanked by one hexagon each side for the biofortification of wheat sprouts with iron (Fe), zinc (Zn), magnesium (Mg), and chromium (Cr), which can effectively stimulate plant growth (right) and by the influence of vanadium (V) in human neurodegenerative disorders such as Alzheimer’s disease (AD), Parkinson’s disease (PD), and amyotrophic lateral sclerosis (ALS) (left).

Data collected in this SI clearly indicate that further studies are needed to better recognize the effects of the interaction between vanadium, of which its pharmacological activity has been repeatedly described [1,2], and other metals, especially those with antioxidant properties [3], as it may be helpful in optimizing the therapeutic potential of vanadium and contribute to the development of effective therapeutic approaches that use this metal in the future. The data also point to the need to better understand the relationships between the structure and properties of metal compounds/complexes, as it may help to explain the modes of their action, thereby making them therapeutically effective. Further studies should also be carried out to define the biological effects of metals/metal-containing species with a special focus on the relationship between the dose and response with the estimation of safe concentrations of the tested metals/metal-containing species at which the potential therapeutic effects will be observed. It is also important to recognize the pharmacokinetic and pharmacodynamic properties of metals and metal-containing compounds/complexes and to clarify the mechanisms of their action in detail. This is crucial in view of the development of metallodrugs in general.

Moreover, there is great interest in repurposing medicines, allowing ‘older’ medicines with marketing approval, such as metformin, to be made available to patients in a timely manner as ‘new’ treatments [4]. Recently, it was suggested that decavanadate and metformin-decavanadate decrease the proliferation of melanoma cells after treatment with these compounds [5]. It was also suggested that certain cell signaling pathways important in the development of cancer are altered by treatment with decavanadate, and how it alters the cell cycle of melanoma cells. Taken together, several studies demonstrate that these applications of polyoxometalates (POMs) in the treatment of cancer [6,7], and specifically vanadium and polyoxovanadates (POVs) in melanoma [5,8], are possible, suggesting a therapeutic opportunity, and strengthening the potential use of these potential metallopharmaceuticals in the near future as anticancer agents, among others biomedical applications such as the understanding of human neurodegenerative disorders [7,8,9,10,11,12,13,14], [contribution 1].

## 2. An Overview of Published Articles

The first paper published in this Special Issue entitled ”CNS-Related Effects Caused by Vanadium at Realistic Exposure Levels in Humans: A Comprehensive Overview Supplemented with Selected Animal Studies” [contribution 1] is an attempt to provide thorough knowledge on the influence of vanadium on neurodegeneration in humans. Hence, it summarizes data on the neurological side effects and neurobehavioral alterations in subjects in relation to vanadium exposure and collects information about other adverse health outcomes of environmental exposure to this metal in humans and findings on the neurotoxic effects of vanadium after inhalation in laboratory animals and those naturally exposed to urban air. It also compiles data on the levels of vanadium in the biological fluids and brain of subjects with neurodegenerative disorders such as AD, PD, and ALS and briefly summarizes new therapies for these illnesses. Data overviewed in this review clearly indicate that more attention should be paid to vanadium, which is a well-known transition metal capable of disturbing the oxidation–reduction balance in the organism, as an environmental risk factor for the health of the general population and to its role in the etiopathogenesis of neurodegenerative diseases in which oxidative stress appears to be a part of the pathophysiological mechanism. They also suggest that vanadium could help in identifying people with a higher risk of detrimental effects on the central nervous system and serve as a predictor of some neurodegenerative diseases. However, more extensive epidemiological studies are needed to confirm this statement.

The original articles included in this SI focus on (1) the assessment of the in vitro effect of copper against the toxicity of bis(maltolato)oxovanadium (IV), BMOV [contribution 2], (2) the evaluation of the structure–activity relationship of fatty acid-like platinum [Pt(IV)] prodrug (FALP) derivatives (a class of Pt-based mitochondria-targeting metallodrugs) in terms of anticancer activity and cellular responses [contribution 3], and (3) the assessment of the DNA interaction with a novel copper(II) indenoisoquinoline complex and its cytotoxic activity against five adenocarcinoma cell lines [contribution 4]. Other topics addressed in the articles include in vitro anticancer and antimicrobial activities along with the antioxidant potential of copper(II) complexes and 1-(isoquinolin-3-yl)heteroalkyl-2-ones [contribution 5], the antimicrobial and in vitro antitumor activity of decavanadate-bearing guanidine derivatives [contribution 6], and in vitro computed tomography (CT) imaging properties along with the in vivo potential toxic effects of monolacunary Wells–Dawson polyoxometalate (mono-DW POM) and the mono-WD POM tissue distribution [contribution 7]. Moreover, the antibacterial effect of Zn(II) carbosilane iminopyridine complexes with inorganic ligands was assessed against certain Gram-positive and Gram-negative bacteria [contribution 8]. In addition, a strategy for bioelement supplementations, which can help to solve current problems associated with the deficiency of some macro- and micro-elements in the human body and the related negative health effect, was assessed [contribution 9]. 

The study conducted in an in vitro model on a hepatic cell line (HepG2) by Rivas-García and co-workers [contribution 2] showed that HepG2 cells exposed to BMOV (3 mg V/L) for 32 h reduces cell viability, whereas the treatment of HepG2 cells with CuCl_2_ (3 mg Cu/L) during incubation with BMOV has the opposite effect. In addition, under the 32 h treatment of the cells with CuCl_2_ (3 mg Cu/L) in the presence of BMOV (3 mg V/L), the ND1/ND4 deletion of the mitochondrial DNA and the nuclear damage caused by the BMOV exposure was slightly limited and markedly reduced, respectively. Thus, these findings clearly show that the 32 h incubation of HepG2 cells with CuCl_2_ in the presence of BMOV does not only enhance the cytotoxic response but also protects against BMOV toxicity, thereby indicating that Cu can effectively prevent the harmful effects caused by V. However, as stressed by the authors, there is a need for further studies to better determine the effects resulting from the interactions between V and Cu, as it may be helpful in limiting the toxic effects of V and enhancing the potential therapeutic applications of this element [contribution 2]. 

Another in vitro study carried out on two human cancer cell lines (A2780cis and MDA-MB-231) [contribution 3] demonstrated that the head group modifications of FALPs can markedly affect their cytotoxicity profiles. More precisely, by using two FALP model compounds, the authors revealed that one of them, i.e., the one with hydrophilic modification, was able to readily penetrate cancer cells and mitochondria, subsequently inducing a cascade of events leading to mitochondrial and DNA damage, which effectively eradicated cancer cells. The other model with hydrophobic modification exhibited significantly lower toxicity (notably lower uptake and weaker cellular responses). Thus, the results obtained from this study clearly showed that increased hydrophobicity, as highlighted by the authors, may not necessarily enhance the cellular uptake of therapeutic molecules [contribution 3]. These findings open a way for new drug development strategies. 

The findings obtained from the in vitro study on human cell lines from cervix cancer, breast cancer, breast triple-negative cancer, colorectal cancer, and prostate cancer (i.e., HeLa, MCF-7, MDA-MB-231, HT-29, and DU-145) conducted by Molinaro and co-workers [contribution 4] showed that the new organometallic compound, i.e., Cu(II)-indenoisoquinoline complex (WN198), can inhibit topoisomerase I in a dose-dependent manner, starting at 1 μM and with optimum efficiency at 2 μM, and that this complex triggers autophagy evidenced by Beclin-1 accumulation and LC3-II formation. This study also demonstrated that WN198 is able to bind to DNA by intercalation, as shown by the melting curves and fluorescence measurements, where the main interaction took place via the aromatic ring. Based on these results, the authors suggest that Cu-derived indenoisoquinoline topoisomerase I inhibitor WN198 may be a promising antitumorigenic agent for the development of future DNA-damaging treatments and emphasize the need for determining the toxicity characteristic of this Cu complex with respect to membrane permeability and cellular uptake. In turn, the study conducted by Balewski and co-workers [contribution 5] on other Cu(II) complexes derived from 1-(isoquinolin-3-yl)heteroalkyl-2-one ligands (i.e., 1-(isoquinolin-3-yl)azetidin-2-one, 1-(isoquinolin-3-yl)imidazolidin-2-one, 1-(isoquinolin-3-yl)-3-methylimidazolidin-2-one, and 1-ethyl-3-(isoquinolin-3-yl)-3-imidazolidin-2-one, L1-4, respectively) evaluated anticancer activity against human cancer A375 (melanoma), HepG2 (hepatoma), LS-180 (colon cancer), and T98C (glioblastoma) cell lines and demonstrated that Cu complexes such as dichloro{bis [1-(isoquinolin-3-yl)azetidin-2-one]}Cu(II) (C1), dichloro{bis [1-(isoquinolin-3-yl)imidazolidin-2-one]}Cu(II) (C2), dichloro [1-(isoquinolin-3-yl)-3-methylimidazolidin-2-one]}Cu(II) (C3), and dichloro [1-ethyl-3-(isoquinolin-3-yl)imidazolidin-2-one]}Cu(II) (C4) have greater potency against HepG2, LS-180, and T98C cells than the known antitumor agent *etoposide*, whereas free ligands L1-4 are inactive in all investigated cell lines. Moreover, the authors showed that dichloro{bis [1-(isoquinolin-3-yl)imidazolidin-2-one]}Cu(II) compound (C2) is more selective towards cancers cells, compared with the non-cancerous human normal skin fibroblasts (CCD-1059sK), than compounds C1, C3, and C4. In addition, they found that the treatment of HepG2 and T98G cells with the copper C2 compound at a concentration that did not inhibit cancer cell growth resulted in an increase in the cytotoxic effects of chemotherapeutics such as *etoposide*, *5-fluorouracil*, and *temozolomide*. Furthermore, microbiological tests, in which the activity of L2-4 ligands and their Cu compounds (C2-4) were evaluated, showed that only L3 exhibited moderate anti-*Candida* activity, while antiradical tests revealed that only the copper C4 complex exhibited the strongest antioxidant potential. Further, the in vitro antitumor and antimicrobial activity was also examined by Dumitrescu’s research team [contribution 6] with respect to decavanadate (DV)-bearing guanidine derivatives (DV-GDs). The isopolyoxovanadate decavanadate (V_10_) and its derivatives are perhaps the most widely studied POV in biology, affecting key biochemical and cellular processes and showing several biomedical applications [4,5,7,8,9,10,11,12,14]. The authors showed that all investigated compounds, i.e., (Hpbg)_4_[H_2_V_10_O_28_]·6H_2_O (DV-GD1), (Htbg)_4_[H_2_V_10_O_28_]·6H_2_O (DV-GD2), (Hgnd)_2_(Hgnu)_4_[V_10_O_28_] (DV-GD3), and (Hgnu)_6_[V_10_O_28_]·2H_2_O (DV-GD4), inhibited the growth of some Gram-negative and Gram-positive bacteria, and DV-GD3 was the most active of these complexes. As far as the cytotoxicity assayed against A375 human melanoma cells and BJ human fibroblasts is concerned, the authors found that all compounds exhibited high cytotoxicity against both melanoma and fibroblast cells, but DV-GD1 was the most active in cancer cells.

Another study included in this SI presents the results of an experiment on monolacunary Wells–Dawson polyoxometalate (mono-WD POM), in which the authors evaluated the in vitro CT imaging properties, in vivo potential toxic effects, and tissue distribution of α_2_-K_10_P_2_W_17_O_61_·20H_2_O [contribution 7]. As outlined in the introduction, POMs have been described as compounds with emergent biomedical activities, as well as being well known to target several cellular key proteins and biological processes [7,8,9,10]. In this study, they found that the acute oral and intravenous administration of mono-WD POM did not induce either physical changes or mortality in rats. As suggested, the lack of mortality and physical alterations noted after the acute oral administration could be linked with the low absorption rate of mono-WD POM due to its large molecular weight. As emphasized, the low bioavailability of POM-based compounds and thus their low toxicity after *per os* treatment indicates that they could be considered as promising candidates for the development of a new oral CT contrast agent. In turn, a good survival rate was reached after intravenous administration, as stressed by the investigators, which indicates that mono-DW POM could also be developed as a new intravenous CT agent. However, the dose-dependent side effects confirmed by biochemical and histological analysis indicate that further studies are needed to develop safe CT contrast agents.

Another study refers to antibacterial activity of Schiff base Zn(II) complexes with three different inorganic ligands, i.e., chloride, nitrate, and acetate (G0[NCPh(*_0_*-N)ZnCl_2_·2H_2_O], G0[NCPh(*_0_*-N)Zn(NO_3_)_2_·2H_2_O, and G0[NCPh(*_0_*-N)Zn(O_2_CCH_3_)_2_, respectively) [contribution 8]. The authors showed that these complexes exhibit moderate antibacterial activity against planktonic bacterial cells of *Staphylococcus aureus* and *Escherichia coli* strains and that this antibacterial effect resulted from the metal complexation to the Schiff base ligand. On the other hand, they found that the impact of the inorganic ligands was not significant for the antibacterial effect but was important for complex solubility. The obtained findings suggest that Zn(II) iminopyridine complexes could be promising candidates for antibacterial therapy.

Finally, the study on the UV-C seed surface sterilization and biofortification of wheat sprouts with iron (Fe), zinc (Zn), magnesium (Mg), and chromium (Cr) [contribution 9] showed that UV-C radiation is effective in preventing infections during seed germination and does not reduce either the growth or development of sprouts or nutrient bioassimilation. Thus, the results of this study clearly showed that irradiation by UV-C light can effectively stimulate plant growth.

## 3. Conclusions and Outlook

The studies described in this SI have provided valuable information about some pharmacologically interesting metals and metal-containing compounds/complexes that were examined for their potential therapeutic uses and as possible CT contrast agents for clinical application. The findings obtained from these studies may lay the groundwork for future research on new therapeutics based on metal-containing compounds/complexes that may ensure better treatment results and guarantee potential clinical success. Additionally, the results of the study focused on the UV-C seed surface sterilization and biofortification of wheat sprouts with certain elements as an effective strategy for bioelement supplementations revealed that irradiation by UV-C light could be used as an effective tool to obtain fortified food in a cheap and quick method.

We believe that the information provided in this SI will appeal to readers who are interested in metals and metal-containing compounds/complexes, as well as human health and nutrition in general, and to those interested in metal interactions and diseases such as cancer, AD, PD, and ALS, which constitute one of the single most important public health challenges.

## References

[1] Ścibior A., Pietrzyk Ł., Plewa Z., Skiba A. (2020). Vanadium: Risks and possible benefits in the light of a comprehensive overview of its pharmacotoxicological mechanisms and multi-applications with a summary of further research trends. J. Trace Elem. Med. Biol..

[2] Ścibior A. (2022). Overview of research on vanadium-quercetin complexes with a historical outline. Antioxidants.

[3] Ścibior A. (2016). Vanadium (V) and magnesium (Mg)—In vivo interactions: A review. Chem. Biol. Int..

[4] De Sousa-Coelho A.L., Fraqueza G., Aureliano M. (2024). Repurposing therapeutic drugs complexed to vanadium in cancer. Pharmaceuticals.

[5] De Sousa-Coelho A.L., Aureliano M., Fraqueza G., Serrão G., Gonçalves J., Sánchez-Lombardo I., Link W., Ferreira B.I. (2022). Decavanadate and metformin-decavanadate effects in human melanoma cells. J. Inorg. Biochem..

[6] Čolović M.B., Lacković M., Lalatović J., Mougharbel A.S., Kortz U., Krstić D.Z. (2020). Polyoxometalates in Biomedicine: Update and Overview. Curr. Med. Chem..

[7] Sánchez-Lara E., Treviño S., Sánchez-Gaytán B.L., Sánchez-Mora E., Eugenia Castro M., Meléndez-Bustamante F.J., Méndez-Rojas M.A., González-Vergara E. (2018). Decavanadate Salts of Cytosine and Metformin: A Combined Experimental-Theoretical Study of Potential Metallodrugs against Diabetes and Cancer. Front. Chem..

[8] Amante C., De Sousa-Coelho A.L., Aureliano M. (2021). Vanadium and melanoma: A systematic review. Metals.

[9] Aureliano M., Gumerova N.I., Sciortino G., Garribba E., Rompel A., Crans D.C. (2021). Polyoxovanadates with emerging biomedical activities. Coord. Chem. Rev..

[10] Aureliano M., Gumerova N.I., Sciortino G., Garribba E., McLauchlan C.C., Rompel A., Crans D.C. (2022). Polyoxidovanadates’ interactions with proteins: An overview. Coord. Chem. Rev..

[11] Carvalho F., Aureliano M. (2023). Polyoxometalates impact as anticancer agents. Int. J. Mol. Sci..

[12] Aureliano M., De Sousa-Coelho A.L., Dolan C.C., Roess D.A., Crans D.C. (2023). Biological Consequences of Vanadium Effects on Formation of Reactive Oxygen Species and Lipid Peroxidation. Int. J. Mol. Sci..

[13] Ferretti V., León I. (2022). An Overview of Vanadium and Cell Signaling in Potential Cancer Treatments. Inorganics.

[14] Gonzalez-Cano S.I., Flores G., Guevara J., Morales-Medina J.C., Treviño S., Diaz A. (2024). Polyoxidovanadates a new therapeutic alternative for neurodegenerative and aging diseases. Neural Regen. Res..

